# Supplementary material for: Impulsivity facets and substance use involvement: insights from genomic structural equation modeling
Source: Psychol Med. 2025 Feb 17;55:e51. doi: 10.1017/S0033291725000145 (PMC12039315; doi:10.1017/S0033291725000145)
Supplement: Vilar-Ribó et al. supplementary material 1 — Vilar-Ribó et al. supplementary material [file S0033291725000145sup001.docx]

**Supplementary Figure 1.** Path diagrams for the confirmatory factor models of impulsivity (**A**) and substance use-related measures (**B**). Observed indicators are represented by squares and latent factors are represented by circles. Single headed arrows indicate factor loading, and double headed arrows indicate correlations. All values indicate standardized parameters estimates.
